# Increased Alpha-Rhythm Dynamic Range Promotes Recovery from Visuospatial Neglect: A Neurofeedback Study

**DOI:** 10.1155/2017/7407241

**Published:** 2017-04-25

**Authors:** Tomas Ros, Abele Michela, Anne Bellman, Philippe Vuadens, Arnaud Saj, Patrik Vuilleumier

**Affiliations:** ^1^Laboratory Behavioral Neurology and Imaging of Cognition, Department of Neuroscience, University Medical Center and Campus Biotech, Geneva, Switzerland; ^2^Romand Clinic of Readaptation, SUVA, Sion, Switzerland; ^3^Neuropsychology Unit, Department of Neurology, University Hospital of Geneva, Geneva, Switzerland

## Abstract

Despite recent attempts to use electroencephalogram (EEG) neurofeedback (NFB) as a tool for rehabilitation of motor stroke, its potential for improving neurological impairments of attention—such as visuospatial neglect—remains underexplored. It is also unclear to what extent changes in cortical oscillations contribute to the pathophysiology of neglect, or its recovery. Utilizing EEG-NFB, we sought to causally manipulate alpha oscillations in 5 right-hemisphere stroke patients in order to explore their role in visuospatial neglect. Patients trained to reduce alpha oscillations from their right posterior parietal cortex (rPPC) for 20 minutes daily, over 6 days. Patients demonstrated successful NFB learning between training sessions, denoted by improved regulation of alpha oscillations from rPPC. We observed a significant negative correlation between visuospatial search deficits (i.e., cancellation test) and reestablishment of spontaneous alpha-rhythm dynamic range (i.e., its amplitude variability). Our findings support the use of NFB as a tool for investigating neuroplastic recovery after stroke and suggest reinstatement of intact parietal alpha oscillations as a promising target for reversing attentional deficits. Specifically, we demonstrate for the first time the feasibility of EEG-NFB in neglect patients and provide evidence that targeting alpha amplitude variability might constitute a valuable marker for clinical symptoms and self-regulation.

## 1. Introduction

Spatial neglect occurs in 30% to 50% of stroke patients [1], characterized by an inability to detect stimuli and orient attention towards the side of space opposite to the lesion [2–4], in the absence elementary sensory or motor deficits, that is, no visual field loss and no paralysis. In addition to severe handicap in daily life for patients and major burden for caregivers, neglect leads to poorer prognosis and reduced benefits from rehabilitation therapies for other neurological deficits [5, 6]. Deepening our knowledge of this syndrome and improving rehabilitation interventions is an important and urgent goal for clinical neuroscience [5]. Here, for the first time, we address this issue by exploiting neurofeedback (NFB): a closed-loop training technique that involves measuring cerebral activity and displaying it in real-time through external feedback, in order to facilitate top-down control of specific activation patterns [7, 8]. NFB training has been successfully applied using either functional MRI or an electroencephalogram (EEG) across different clinical domains [9].

There is convergent evidence from functional imaging studies that neglect entails impaired top-down modulation of visual processing subsequent to brain injury. Not only frontoparietal cortical areas implicated in attentional control show abnormal asymmetries due to lesion or reduced activation in the damaged hemisphere [10, 11], but early visual areas also exhibit attenuated responses to sensory stimuli even when anatomically spared [12]. A restoration of activity is observed in both sensory and parietal areas after neglect recovery [10, 13] or following effective rehabilitation [14, 15].

Consistent with this account, unilateral stroke is reported to produce a baseline reduction of cortical excitability in the affected relative to the unaffected hemisphere [16], which is reversed after recovery or treatment with noninvasive brain stimulation [17]. Specifically, excitability-increasing protocols (e.g., high-frequency TMS, anodal tDCS) to the right posterior parietal cortex (rPPC) of neglect patients is associated with improved perception in the left visual field [18–20]. Hence, training to upregulate rPPC cortical excitability and/or enhancing residual sensory activation through various means [15, 21] might provide an alternate and potentially safer way to restore visuospatial awareness. Indeed, direct stimulation techniques produce electromagnetic fields that are not intrinsic to the brain and thus still need to be validated for their long-term safety, including occasional seizure occurrence [22–24].

Here, we directly tested this hypothesis by deploying EEG-based NFB, whose high temporal resolution, low cost, and accessibility favors direct clinical translation. EEG studies have found that alpha oscillations exhibit a retinotopically selective reduction over parietal areas during attention to visual targets in the contralateral field [25, 26] and successful detection of visual targets is associated with greater reductions of prestimulus alpha amplitude [27, 28]. Secondly, lateralized entrainment of alpha oscillations via TMS causally inhibits visual detection within the contralateral hemifield [29]. Likewise, the degree of pseudoneglect in the healthy population appears to correlate with individual variations in posterior alpha amplitude [30]. These effects may be explained by a mechanism where localized alpha oscillations act as an inhibitory gate for sensory processing [31]. Furthermore, several studies reported that NFB control of parietal alpha oscillations may be mastered by healthy participants [7, 32], thereby altering visuospatial bias [33] and inducing lasting increases in cortical excitability [34].

Based on this knowledge, we recruited 5 stroke patients with left visuospatial neglect, in whom we causally downregulated alpha oscillations with NFB in order to (i) evaluate the feasibility of NFB training in such patients, (ii) assess its potential benefit as a novel target for neglect rehabilitation, and (iii) examine their role in pathological spatial attention subsequent to stroke.

## 2. Methods and Materials

### 2.1. Patients

Patients participated after giving their written informed consent. The study was approved by Geneva State Ethics Committee and accorded with the Helsinki declaration. Patients were admitted after a first right cerebral stroke. We excluded patients with bilateral lesions, previous neurological or psychiatric disorders, impairment in primary visual perception (except partial visual field defect), psychiatric disorders, motor difficulties in the right upper limb, pusher syndrome (i.e., contralateral trunk deviation with active resistance to any attempt of external correction), or current psychotropic treatment. Spatial neglect was assessed using a standard clinical battery similar to other research in our group [15, 35] and diagnosed when patients demonstrated abnormal performance in the following tests: line bisection (cutoff: rightward deviation >11%) [36] and target cancellation test (cutoff: left–right omissions ≥4 out of 15 omissions) [37].

Five right-hemisphere-lesioned participants (mean age: 61, SD: 6, 1 woman, 4 men) fulfilling these criteria were recruited prospectively from the Clinique de Réadaptation of SUVA in Sion (Foundation Valais de Coeur of Sion and Sierre). All underwent structural MRI scans to delineate the location and extent of brain damage (see Supplementary Figure S1 available online at https://doi.org/10.1155/2017/7407241).

### 2.2. Experimental Design

The overall study duration spanned 2 weeks. It consisted of a 1-week waitlist control period (with continued treatment as usual), followed by 1 week of NFB training (6 sessions on a daily basis). The first EEG and behavioral assessment (see Supplementary Figure S2, gold slot) took place at the beginning of the waitlist period (i.e., one week prior to NFB training). This period allowed us to probe for any spontaneous recovery prior to NFB. Patients' EEG and behavior were then retested on the first day of NFB training (Figure S2, brown slots), both before and immediately after the NFB session. An identical pre-to-post NFB assessment occurred during the last (i.e., 6th) day of neurofeedback training.

No adverse effects or unusual symptoms were reported by any patient either before, during, or after the NFB training sessions. This was corroborated by clinical neuropsychologists in charge of the patients.

### 2.3. Clinical Battery for Visuospatial Neglect

A series of standard paper-and-pencil tasks were presented to the participant once at the first visit (prewaitlist, gold slot in Figure S2), twice at the second visit (flanking the 1st NFB session, brown slot in Figure S2), and twice at the last visit (flanking the 6th NFB session, brown slot in Figure S2). This was done in order to quantify patients' baseline as well as short- and long-term changes in visuospatial biases. Neglect severity was measured with the Schenkenberg line bisection task [36] (18 horizontal lines, 10–20 cm) and a variant of the bell cancellation test [37] (with 35 animal targets (among distractor objects)) [38]. Four equivalent animal search displays were used, each divided into seven virtual columns (each containing 5 targets).

### 2.4. EEG Recording and Processing

A multichannel EEG cap was used to measure whole-scalp activity in the resting state in each baseline testing session (Figure S2, gold and brown slots). Scalp voltages were recorded using a 19 Ag/AgCl electrode cap (Electro-Cap International Inc., www.electro-cap.com) according to the 10-20 international system: the ground electrode was placed on the scalp, at a site equidistant between Fpz and Fz. Electrical signals were amplified with the Mitsar 21-channel EEG system (Mitsar-201, CE0537, Mitsar Ltd., http://www.mitsar-medical.com), and all electrode impedances were kept under 5 kΩ. For online recording, electrodes were referenced to linked earlobes, and then the common average reference was calculated offline before further analysis. EEG data was recorded at 250 Hz and then filtered with a 0.5–40 Hz bandpass filter offline.

All EEG data were imported into the Matlab toolbox EEGLAB v12 (http://sccn.ucsd.edu/eeglab/) for offline processing. We used Infomax ICA decomposition to remove usual eye movement such as saccades or blinking [39]. Recordings were further cleaned with an automated *z*-score based method, using the FASTER plugin [40], rejecting 1-second epochs that deviated from the mean by more than 1.7 standard deviations.

### 2.5. Quantitative Markers of EEG Activity

Artifact-free EEG data was analysed with the Neurophysiological Biomarker Toolbox (NBT, http://www.nbtwiki.net/). Given well-known links between alpha activity and visual attention (see Section 1), our analyses focused on the alpha frequency band (7–13 Hz). Here, absolute alpha amplitude was estimated with a standard FFT approach using Welch's method (Matlab *pwelch()* function) and a Hanning windowing function (4-second epoch, 50% overlap). In addition to mean measures, we assessed the variability (i.e., dynamic range) of EEG amplitude in the alpha band by calculating the instantaneous alpha amplitude (envelope) across time using the absolute value of the Hilbert transform, that is, Matlab *hilbert()* function. Alpha variability was then defined using the coefficient of variation *c*_v_, with *σ* (standard deviation) and *μ* (mean).

(1) $$\begin{array}{c} c_{v}=\frac{\sigma }{\mu }. \end{array}$$

Finally, following Thut et al. [41], a posterior interhemispheric imbalance was defined as the left-to-right asymmetry in the aforementioned measures, specifically at parietal electrodes P3‐P4:

(2) $$\begin{array}{c} AS\left(P3,P4\right)=\frac{\left(P3-P4\right)}{\left(P3+P4\right)}. \end{array}$$

Here, values below 1 represent lower alpha amplitude in the right hemisphere relative to the left, whereas values above 1 represent greater alpha amplitude in the right relative to the left hemisphere.

In a second step, we sought to explore group EEG differences between patients and the normative population. EEG recordings from our patients were compared to a control group of 30 healthy adults (mean age: 49, SD: 9, males: 15, females: 15), randomly sampled from the Human Brain Institute (HBI) normative database (http://www.hbimed.com/) [42]. Importantly, the HBI database data was collected using the same amplifier type (Mitsar-201) for recordings of healthy subjects. Topographic (channel-wise) statistical comparisons of EEG markers were then carried in using the NBT Toolbox via independent *t*-tests.

### 2.6. EEG Neurofeedback

Neurofeedback was performed for 6 daily sessions lasting 30 minutes each. Feedback was provided from electrode P4 (right parietal cortex) aimed at downregulation of alpha (7–13 Hz) amplitude, using a similar visual display as our previous studies [7, 34]. Please see Supplementary Results for additional details.

## 3. Results

In the results that follow, we refer to pre-to-post differences flanking the NFB procedure in the first and last training sessions as *short-term effects* (within session) and the longitudinal changes from before to after a week of each intervention (i.e., waitlist and full NFB course) as *long-term effects.*

### 3.1. Neurofeedback Control: Alpha Amplitude *during* the First and Last Sessions

During each training session, patients attempted to gain control of the NFB signal, which consisted of a relative suppression of alpha (7–13 Hz) amplitude at the right parietal cortex. Each 21-minute daily NFB session was subdivided into 7 × 3-minute training runs, preceded by an eyes-open resting state baseline. We first examined the online evolution of alpha amplitude at the right posterior parietal cortex (rPPC) feedback-channel (i.e., P4), during each of these runs (baseline + 21 min regulation), for the first (1st) and last (6th) training sessions. Other sessions (2nd to 5th) comprised similar NFB training runs but no whole-scalp EEG recordings (besides the P4 electrode used for NFB).

**Figure 1:**
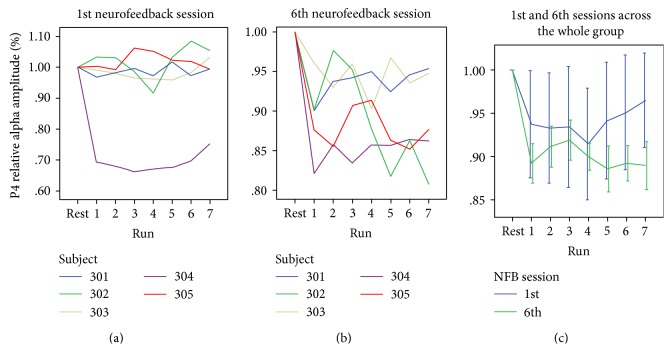
Alpha amplitude dynamics during the first and last sessions of neurofeedback (NFB) (corresponding to brown slots in Figure S2). Each time point represents data acquired before NFB (rest) and during each NFB run of 3 min (runs 1–7). (a) Individual patient values during the 1st NFB session. (b) Individual patient values during the 6th NFB session. (c) Group (*n* = 5) means from the 1st (first) and 6th (last) NFB sessions are depicted in green and blue, respectively.

As shown in Figure 1(a), during the first session, alpha amplitude could be downregulated successfully in one patient only, whereas the last session (Figure 1(b)) demonstrated a groupwise negative trend in % amplitude reduction over successive training runs, with a smaller variability between subjects. An initial two-way ANOVA with the factors session (first or last) and time-bin (baseline and training runs 1–7) did not show a significant interaction (n.s.). However, separate one-way ANOVAs for each session revealed a significant main effect of time-bin for the *last* session only (*F*_7,28_ = 6.3, *p* = 0.01), which was absent in the *first* (*F*_7,28_ = 1.016, n.s.). As seen in Figure 1(c), these data suggest that by the 6th session, the majority of patients were able to exert more pronounced and consistent control of their right parietal activity in the direction of training. Applying the Fisher *r*-to-*z* transformation to the group regression coefficient between alpha amplitude and time-bin, we observed a significant difference (*z* = 1.98, *p* < 0.05) between coefficients of the 1st (*r* = 0.001) and 6th (*r* = −0.43) sessions. This further supports the idea of learned improvement in NFB control across the week of training.

### 3.2. Short- and Long-Term Effects on Resting-State Alpha Rhythm

**Figure 2:**
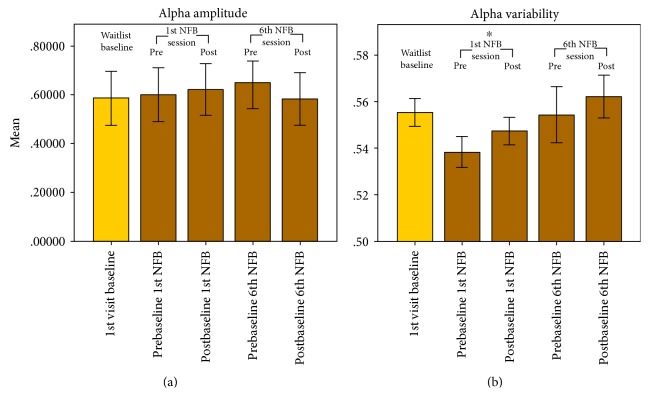
Right posterior parietal cortex (rPPC) resting-state alpha amplitude (a) and variability (b), pre-to-post 1st and 6th NFB sessions (brown), compared to the initial waitlist baseline (gold). Alpha amplitude variability tended to increase pre- to postregulation in the 1st session, whereas mean amplitude decreased pre- to postregulation in the 6th session. ^∗^Significant difference at *p* < 0.05.

In addition to within-session NFB changes *during* the training runs, we investigated the short-term effect of *resting-state* alpha-amplitude as recorded immediately before and after NFB [7, 43].  Figure 2 illustrates the group baseline values taken just before and after the 1st and 6th NFB sessions over the right PPC (i.e., electrode P4).

Pre–post EEG resting measures flanking the 1st NFB session showed a significant increase in alpha variability (*s*, *p* < 0.01), but not amplitude (−0.22, n.s.). Compatible with hypotheses, the last (6th) NFB training session exhibited a *decrease* in alpha amplitude (*t*_4_ = 2.25, one-tailed *p* = 0.04), along with a trend increase in alpha variability (*t* = −1.2, one tailed *p* = 0.15). These findings suggest a promising normalization of alpha dynamic range (i.e., variability) after NFB, compatible with a recent theoretical framework [9]. Following the waitlist period (Figure 2), the alpha amplitude was not significantly altered (*t*_4_ = −0.8, n.s.), while the alpha variability initially decreased (*t*_4_ = 10.3, *p* < 0.01), only to return to similar baseline levels in at the last session of NFB.

### 3.3. Resting-State Alpha Rhythm Comparison to a Control Group of Healthy Older Adults

To clarify whether the main EEG marker(s) modulated by NFB training (e.g., alpha amplitude, alpha variability) moved towards or away from those of the normal population, we compared data from our patients with a control group of 30 healthy older adults sampled from a reference cohort using the same recording methodology [42].

**Figure 3:**
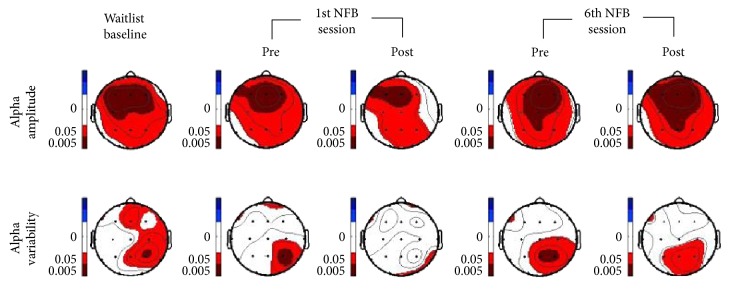
Topographical significance plots for the healthy control group minus patients' values, for alpha amplitude and normalized variability; at baseline, plus before/after the first and last neurofeedback sessions. Red and dark red areas represent group differences with higher values in control > patients, *p* < 0.05 and *p* < 0.005, respectively.

As shown in Figure 3, at the first visit (waitlist) baseline, patients demonstrated a statistically significant reduction of alpha amplitude over the whole scalp, in line with previous EEG studies in stroke and hemineglect [44, 45]. Interestingly, these global differences relative to the control group were most pronounced within the frontal lobes (*p* < 0.005). More notably, patients also exhibited a prominent and markedly asymmetric decrease of alpha variability, whose focus was centered around the rPPC (P4 electrode, *p* < 0.005), coinciding with the site of the brain-behavior relationships typically observed in spatial neglect [46, 47].

Following the first session of NFB training, the extent of alpha amplitude difference (marked dark red) in the patients compared to healthy controls only slightly diminished. Crucially, in contrast, alpha variability effectively normalized to values similar to the control group at the P4 electrode, mostly reversing the initial significant differences (n.s.). Likewise, following the last NFB session, as patients reduced their alpha rhythm more consistently during the regulation runs, this did not translate into significant differences in mean alpha amplitude at rest over the frontal lobe (with equal or even greater differences in comparison with the control group means, depicted by a large spreading of the dark red area, *p* < 0.005, Figure 3). Again, however, the alpha variability was significantly increased post-NFB (*p* > 0.01) towards healthy control group values, specifically over the right parietal areas. These results point to the significance of alpha variability at rest as a functional biomarker of pathological neural activity in neglect patients.

### 3.4. Within-Session NFB Training Dynamics Predict Short-Term Effects on Visuospatial Bias

A functional link between alpha-rhythm dynamics and spatial neglect was also more directly examined, first by testing for any correlation between alpha amplitude modulation during NFB and changes in attentional performance pre-to-post a single session of NFB. Here, for each individual patient, we correlated the relative change in alpha amplitude during neurofeedback (expressed as a % change from resting state) with the pre–post scores on clinical neglect tests (line bisection test, target cancellation test). As shown in Table 1 for the cancellation test, already at the first NFB session, alpha amplitude change during NFB positively predicted the reduction of omissions to the left (*r* = 0.88, *p* = 0.05) and center (*r* = 0.96, *p* < 0.05) of the display. In other words, successful decreases in alpha amplitude were associated with lower omission rates pre-to-post NFB, in line with initial hypotheses about the NFB protocol.

In contrast, we did not observe any such significant correlations for the 6th (last) NFB session. This could potentially be due to the relative normalization of individual alpha desynchronization by the 6th session (see Figure 1), reducing the magnitude of amplitude differences during regulation.

### 3.5. Longitudinal Correlations between Visuospatial Performance and Resting-State Alpha Rhythm

Next, we investigated whether EEG and behavioral parameters would coevolve along longer timescales, now conducting correlation analyses longitudinally across all time points of testing (yellow and brown slots, see Figure S2). Here, the measures were converted to *z*-scores, with the first time point being the resting state baseline EEG from the first waitlist visit, the second and third were the pre- and postbaselines flanking the first NFB session, while the fourth and fifth were the baselines flanking the last NFB training session. For the mean alpha amplitude at P4 (the NFB controlled parameter), we found no significant correlations with any neglect severity measures (i.e., omissions on the left, center, or right parts of the cancellation test, deviation on line bisection). However, as shown in Table 2, for the alpha *variability* and its left–right parietal asymmetry, we observed significant correlations with performance on the cancellation test.

As shown in Table 2, alpha variability at the rPPC as well as its hemispheric asymmetry (lPPC-rPPC) correlated negatively with the total omission levels (rPPC: *r* = −0.49, *p* = 0.019; asymmetry: *r* = 0.58, *p* = 0.004) as well as center omissions (rPPC: *r* = −0.45, *p* = 0.03; asymmetry: *r* = 0.58, *p* = 0.004) on the cancellation test. The correlation with left-sided omissions was marginal for alpha variability values (*r* = −0.382, *p* = 0.072), but statistically significant for its asymmetry (*r* = 0.51, *p* = 0.01). In contrast, omission rates on the right side were uncorrelated to either of these EEG markers.

**Figure 4:**
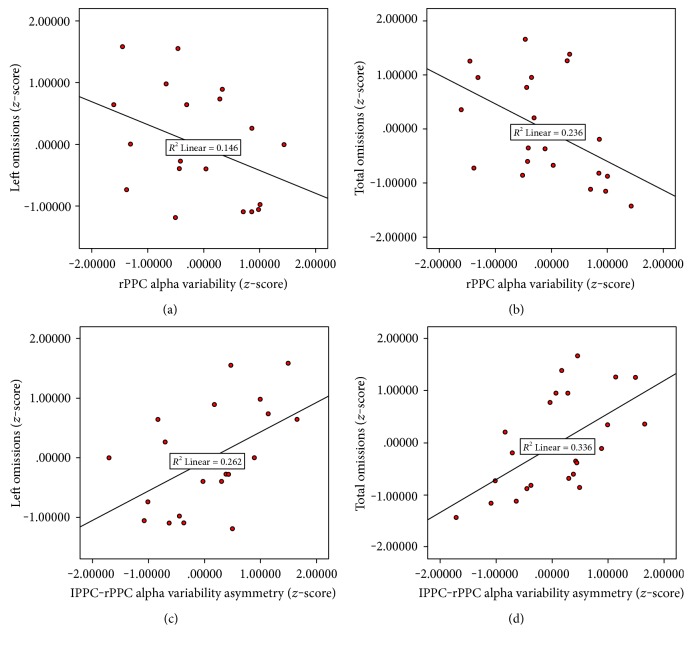
Scatter plots showing the alpha variability over the right parietal cortex (panels (a) and (b)) and its interhemispheric asymmetry (panels (c) and (d)) as a function of omission rates in the cancellation test. Each dot represents individual patient data from 5 resting-state baseline measures (waitlist and before/after NFB training).

Scatter plots of these significant correlations are depicted in Figure 4. Negative and positive correlations indicate that omission rates decreased with increased alpha dynamic range over the rPPC, but increased with greater rPPC-lPPC asymmetry. Hence, interhemispheric variability explained more behavioral covariance compared to rPPC alone. Importantly, correlations were significant for left or center omission rates (but not right omissions), making them relatively specific for the typical behavioral deficit observed in spatial neglect.

## 4. Discussion

We should acknowledge at the outset the principal limitation of our study: due to its small sample size and low statistical power, we cannot fully exclude the possibility that behavioral improvements seen in our patients were partly due to time and/or habituation to the tasks (see Supplementary Figure S3). However, this seems unlikely given the observed dynamics of alpha activity over training sessions and their relation to neglect symptoms. To our knowledge, this is the first published study to apply NFB to neglect rehabilitation, despite the limited therapeutic tools in this condition. Further, few studies used NFB with real-time EEG [48, 49] or fMRI [50] in stroke patients, with similar or even smaller sample size than our study. Hence, our preliminary findings provide a novel proof of principle concerning the feasibility of NFB in neglect patients and potential neurophysiological markers associated with visuospatial attention deficits.

A first positive result was that neglect patients could learn control over the EEG neurofeedback parameter over time (i.e., alpha-band reduction at rPPC). Indeed, a reduction of alpha amplitude below resting-state baseline achieved statistical significance during the NFB runs in the last compared to the first session, supporting the notion that NFB training might be effective in this population.

Support for a NFB-related improvement emerged from a convergence of findings. Firstly, we observed that error reduction in the cancellation test was predicted by the very parameter *trained during the 1st session of NFB* (i.e., the relative degree of alpha amplitude reduction). In healthy individuals, alpha amplitude reductions were found to predict successful sensory detection [51, 52], during which the excitability [53] and neuronal spike rate [52] of sensory cortex is heightened. Conversely, attentional lapses coincide with increases in alpha amplitude [54]. Recent theoretical frameworks [31, 55] proposed that higher alpha synchronization reflects inhibition of sensory cortical areas [52, 53]. Moreover, there is abundant evidence linking spontaneous fluctuations of alpha amplitude with target processing in the contralateral visual hemifield [26, 33, 56, 57], as well as a remarkable correspondence between retinotopic locations and alpha scalp topography [58].

Secondly, by examining the EEG correlates of left visuospatial recovery across all 5 assessments, we found that resting-state alpha amplitude *variability* at rPPC (and rPPC-lPPC asymmetry) was the principal predictor of symptom change. Not only was alpha variability markedly asymmetric over the posterior scalp in patients relative to healthy controls (see Figure 3), but this asymmetry was also consistently reduced after NFB training (Figure 3) and furthermore correlated with changes in neglect symptoms in cancellation performance (Figure 4).

Intriguingly, alpha amplitude pre-to-post NFB in the 1st session produced no significant changes, whereas the 6th (last) session showed a hypothesized decrease (see also Ros et al. [9]). This was echoed by a significant increase in dynamic range (alpha variability) after the 1st NFB session, but not the 6th session. Similar “rebound effects” have been documented in psychiatric populations, after single [43] or repeated [9] NFB sessions. As argued in a recent theoretical framework [9], NFB might restore mechanisms of network homeostasis, which maintained an abnormal state of excitation-inhibition (*E*/*I*) balance [34, 59] due to focal damage or (mal)adaptive plasticity. Abnormal baseline values of alpha amplitude (extending globally) and its dynamic range (prevalent over rPPC) may provide a neurophysiological marker of spatial neglect indexing *E*/*I* deviations and alterations in the functional dynamics of attentional networks.

Overall, besides demonstrating for the first time the feasibility of EEG NFB in neglect patients, our study provides preliminary evidence that targeting *alpha amplitude variability* might constitute a valuable marker for clinical symptoms and self-regulation. Future studies might consider exploring the role of other oscillatory measures sensitive to attention deficits and neural dysfunctions following ischemic stroke [44, 45], preferably administrating NFB training also to healthy participants, in order to test for the specificity of changes to pathological EEG rhythms [60].

## Supplementary Material

Figure S1. Individual structural MRI lesion maps of the 5 neglect patients. Figure S2. Experimental timeline for each patient. Gold: Initial baseline assessment consisting of resting-state EEG and behavioral tests, followed by a wait-list period (Week 1). Brown: Pre-post baseline assessment flanking first and last session of NFB training. Green: NFB training only. Patients underwent 21 minutes of neurofeedback training on a daily basis (except weekends). Figure S3. Omission rates for the target cancellation test at the 5 different times of assessment (2nd and 3rd, as well as 4th and 5th are pre-to-post NFB measures). Omission rate is plotted for A) the entire search display (total), B) center, C) left , and D) right side.

## Figures and Tables

**Table 1:** Correlation between NFB modulation of alpha amplitude (at electrode P4) and changes in errors of omission in the target cancellation test, for the (a) 1st NFB session and (b) 6th NFB session.

Target cancellation test	Alpha amplitude during NFB (% change)	
	1st NFB session	6th NFB session
Total omissions: pre–post NFB (% change)	*r* = 0.87 *p* = 0.055	*r* = −0.14 *p* = 0.85
Left omissions: pre–post NFB (% change)	*r* = 0.88^∗^ *p* = 0.047	*r* = 0.14 *p* = 0.86
Right omissions: pre–post NFB (% change)	*r* = 0.25 *p* = 0.75	*r* = 0.01 *p* = 0.95
Center omissions: pre–post NFB (% change)	*r* = 0.96^∗^ *p* = 0.038	*r* = 0.73 *p* = 0.27

^∗^Significant correlation at *p* < 0.05

**Table 2:** Correlations of alpha variability (rPPC and lPPC-rPPC asymmetry) with behavioral performance on the target cancellation test, as well as left error rate in the line bisection test.

	rPPC Alpha variability	lPPC-rPPC Alpha variability asymmetry
Cancellation test: total omissions	*r* = −0.49 *p* = 0.02	*r* = 0.58 *p* = 0.004
Cancellation test: left omissions	*r* = −0.38 *p* = 0.07	*r* = 0.51 *p* = 0.01
Cancellation test: center omissions	*r* = −0.45 *p* = 0.03	*r* = 0.58 *p* = 0.004
Cancellation test: right omissions	*r* = −0.14 *p* = 0.52	*r* = 0.28 *p* = 0.20
Line bisection test: left deviation error	*r* = −0.40 *p* = 0.06	*r* = 0.47 *p* = 0.02

## References

[1] Appelros P., Karlsson G. M., Seiger A., Nydevik I. (2002). Neglect and anosognosia after first-ever stroke: incidence and relationship to disability. *Journal of Rehabilitation Medicine*.

[2] Corbetta M., Shulman G. L. (2011). Spatial neglect and attention networks. *Annual Review of Neuroscience*.

[3] Nijboer T., van de Port I., Schepers V., Post M., Visser-Meily A. (2013). Predicting functional outcome after stroke: the influence of neglect on basic activities in daily living. *Frontiers in Human Neuroscience*.

[4] Vuilleumier P. O., Rafal R. D. (2000). A systematic study of visual extinction. Between- and within-field deficits of attention in hemispatial neglect. *Brain*.

[5] Chen P., Hreha K., Kong Y., Barrett A. M. (2015). Impact of spatial neglect on stroke rehabilitation: evidence from the setting of an inpatient rehabilitation facility. *Archives of Physical Medicine and Rehabilitation*.

[6] Nys G. M. S., van Zandvoort M. J. E., de Kort P. L. M. (2005). The prognostic value of domain-specific cognitive abilities in acute first-ever stroke. *Neurology*.

[7] Ros T., Théberge J., Frewen P. A. (2013). Mind over chatter: plastic up-regulation of the fMRI salience network directly after EEG neurofeedback. *NeuroImage*.

[8] Robineau F., Rieger S. W., Mermoud C. (2014). Self-regulation of inter-hemispheric visual cortex balance through real-time fMRI neurofeedback training. *NeuroImage*.

[9] Ros T., Baars B. J., Lanius R. A., Vuilleumier P. (2014). Tuning pathological brain oscillations with neurofeedback: a systems neuroscience framework. *Frontiers in Human Neuroscience*.

[10] Corbetta M., Kincade M. J., Lewis C., Snyder A. Z., Sapir A. (2005). Neural basis and recovery of spatial attention deficits in spatial neglect. *Nature Neuroscience*.

[11] Umarova R. M., Saur D., Kaller C. P. (2011). Acute visual neglect and extinction: distinct functional state of the visuospatial attention system. *Brain*.

[12] Vuilleumier P. (2013). Mapping the functional neuroanatomy of spatial neglect and human parietal lobe functions: progress and challenges. *Annals of the new York Academy of Sciences*.

[13] Thimm M., Fink G. R., Sturm W. (2008). Neural correlates of recovery from acute hemispatial neglect. *Restorative Neurology and Neuroscience*.

[14] Thimm M., Fink G. R., Küst J., Karbe H., Willmes K., Sturm W. (2009). Recovery from hemineglect: differential neurobiological effects of optokinetic stimulation and alertness training. *Cortex*.

[15] Saj A., Cojan Y., Vocat R., Luauté J., Vuilleumier P. (2013). Prism adaptation enhances activity of intact fronto-parietal areas in both hemispheres in neglect patients. *Cortex*.

[16] Fregni F., Pascual-Leone A. (2007). Technology insight: noninvasive brain stimulation in neurology-perspectives on the therapeutic potential of rTMS and tDCS. *Nature Clinical Practice. Neurology*.

[17] Fregni F., Boggio P. S., Valle A. C. (2006). A sham-controlled trial of a 5-day course of repetitive transcranial magnetic stimulation of the unaffected hemisphere in stroke patients. *Stroke*.

[18] Sunwoo H., Kim Y. H., Chang W. H., Noh S., Kim E. J., Ko M.-H. (2013). Effects of dual transcranial direct current stimulation on post-stroke unilateral visuospatial neglect. *Neuroscience Letters*.

[19] Sparing R., Thimm M., Hesse M. D., Küst J., Karbe H., Fink G. R. (2009). Bidirectional alterations of interhemispheric parietal balance by non-invasive cortical stimulation. *Brain*.

[20] Roy L. B., Sparing R., Fink G. R., Hesse M. D. (2015). Modulation of attention functions by anodal tDCS on right PPC. *Neuropsychologia*.

[21] Domínguez-Borràs J., Armony J. L., Maravita A., Driver J., Vuilleumier P. (2013). Partial recovery of visual extinction by pavlovian conditioning in a patient with hemispatial neglect. *Cortex*.

[22] Nitsche M. A. (2015). Co-incidence or causality? Seizures after slow rTMS in stroke patients. *Clinical Neurophysiology*.

[23] Davis N. J., van Koningsbruggen M. G. (2013). ‘Non-invasive’ brain stimulation is not non-invasive. *Frontiers in Systems Neuroscience*.

[24] Pelletier S. J., Cicchetti F. (2014). Cellular and molecular mechanisms of action of transcranial direct current stimulation: evidence from in vitro and in vivo models. *The International Journal of Neuropsychopharmacology*.

[25] Rihs T. A., Michel C. M., Thut G. (2007). Mechanisms of selective inhibition in visual spatial attention are indexed by alpha-band EEG synchronization. *The European Journal of Neuroscience*.

[26] Cosmelli D., López V., Lachaux J. P. (2011). Shifting visual attention away from fixation is specifically associated with alpha band activity over ipsilateral parietal regions. *Psychophysiology*.

[27] Wyart V., Tallon-Baudry C. (2009). How ongoing fluctuations in human visual cortex predict perceptual awareness: baseline shift versus decision bias. *The Journal of Neuroscience*.

[28] Hanslmayr S., Aslan A., Staudigl T., Klimesch W., Herrmann C. S., Bäuml K. H. (2007). Prestimulus oscillations predict visual perception performance between and within subjects. *NeuroImage*.

[29] Romei V., Gross J., Thut G. (2010). On the role of prestimulus alpha rhythms over occipito-parietal areas in visual input regulation: correlation or causation?. *The Journal of Neuroscience*.

[30] Cicek M., Nalcaci E., Kalaycioglu C. (2009). Line bisection task performance and resting EEG alpha power. *The International Journal of Neuroscience*.

[31] Jensen O., Mazaheri A. (2010). Shaping functional architecture by oscillatory alpha activity: gating by inhibition. *Frontiers in Human Neuroscience*.

[32] Horschig J. M., Oosterheert W., Oostenveld R., Jensen O. (2014). Modulation of posterior alpha activity by spatial attention allows for controlling a continuous brain-computer interface. *Brain Topography*.

[33] Okazaki Y. O., Horschig J. M., Luther L., Oostenveld R., Murakami I., Jensen O. (2015). Real-time MEG neurofeedback training of posterior alpha activity modulates subsequent visual detection performance. *NeuroImage*.

[34] Ros T., Munneke M., Ruge D., Gruzelier J., Rothwell J. (2010). Endogenous control of waking brain rhythms induces neuroplasticity in humans. *The European Journal of Neuroscience*.

[35] Vaessen M. J., Saj A., Lovblad K. O., Gschwind M., Vuilleumier P. (2016). Structural white-matter connections mediating distinct behavioral components of spatial neglect in right brain-damaged patients. *Cortex*.

[36] Schenkenberg T., Bradford D. C., Ajax E. T. (1980). Line bisection and unilateral visual neglect in patients with neurologic impairment. *Neurology*.

[37] Gauthier L., Dehaut F., Joanette Y. (1989). The bells test: a quantitative and qualitative test for visual neglect. *International Journal of Clinical Neuropsychology*.

[38] Gassama S., Deplancke A., Saj A., Honoré J., Rousseaux M. (2011). Do supine position and deprivation of visual environment influence spatial neglect?. *Journal of Neurology*.

[39] Jung T. P., Makeig S., Humphries C. (2000). Removing electroencephalographic artifacts by blind source separation. *Psychophysiology*.

[40] Nolan H., Whelan R., Reilly R. B. (2010). FASTER: fully automated statistical thresholding for EEG artifact rejection. *Journal of Neuroscience Methods*.

[41] Thut G., Nietzel A., Brandt S. A., Pascual-Leone A. (2006). α-Band electroencephalographic activity over occipital cortex indexes visuospatial attention bias and predicts visual target detection. *The Journal of Neuroscience*.

[42] Grin-Yatsenko V. A., Baas I., Ponomarev V. A., Kropotov J. D. (2009). EEG power spectra at early stages of depressive disorders. *Journal of Clinical Neurophysiology*.

[43] Kluetsch R. C., Ros T., Théberge J. (2014). Plastic modulation of PTSD resting-state networks and subjective wellbeing by EEG neurofeedback. *Acta Psychiatrica Scandinavica*.

[44] Finnigan S., van Putten M. J. a. M., Van Putten M. J. a. M. (2012). Clinical neurophysiology EEG in ischaemic stroke: quantitative EEG can uniquely inform (sub-)acute prognoses and clinical management. *Clinical Neurophysiology*.

[45] Demeurisse G., Hublet C., Paternot J. (1998). Quantitative EEG in subcortical neglect. *Neurophysiologie Clinique*.

[46] Colson C., Demeurisse G., Hublet C., Slachmuylder J. L. (2001). Subcortical neglect as a consequence of a remote parieto-temporal dysfunction. A quantitative EEG study. *Cortex*.

[47] Nyffeler T., Cazzoli D., Wurtz P. (2008). Neglect-like visual exploration behaviour after theta burst transcranial magnetic stimulation of the right posterior parietal cortex. *The European Journal of Neuroscience*.

[48] Young B. M., Nigogosyan Z., Walton L. M. (2014). Changes in functional brain organization and behavioral correlations after rehabilitative therapy using a brain-computer interface. *Frontiers in Neuroengineering*.

[49] Várkuti B., Guan C., Pan Y. (2013). Resting state changes in functional connectivity correlate with movement recovery for BCI and robot-assisted upper-extremity training after stroke. *Neurorehabilitation and Neural Repair*.

[50] Sitaram R., Veit R., Stevens B. (2012). Acquired control of ventral premotor cortex activity by feedback training: an exploratory real-time fMRI and TMS study. *Neurorehabilitation and Neural Repair*.

[51] Ergenoglu T., Demiralp T., Bayraktaroglu Z., Ergen M., Beydagi H., Uresin Y. (2004). Alpha rhythm of the EEG modulates visual detection performance in humans. *Cognitive Brain Research*.

[52] Haegens S., Nacher V., Luna R., Romo R., Jensen O. (2011). Oscillations in the monkey sensorimotor network influence discrimination performance by rhythmical inhibition of neuronal spiking. *Proceedings of the National Academy of Sciences*.

[53] Romei V., Brodbeck V., Michel C., Amedi A., Pascual-Leone A., Thut G. (2008). Spontaneous fluctuations in posterior ??-band EEG activity reflect variability in excitability of human visual areas. *Cerebral Cortex*.

[54] Huang R. S., Jung T. P., Delorme A., Makeig S. (2008). Tonic and phasic electroencephalographic dynamics during continuous compensatory tracking. *NeuroImage*.

[55] Klimesch W., Sauseng P., Hanslmayr S. (2007). EEG alpha oscillations: the inhibition-timing hypothesis. *Brain Research Reviews*.

[56] Dombrowe I., Hilgetag C. C. (2014). Occipitoparietal alpha-band responses to the graded allocation of top-down spatial attention. *Journal of Neurophysiology*.

[57] Ikkai A., Dandekar S., Curtis C. E. (2016). Lateralization in alpha-band oscillations predicts the locus and spatial distribution of attention. *PloS One*.

[58] Rihs T. A., Michel C. M., Thut G. (2009). A bias for posterior alpha-band power suppression versus enhancement during shifting versus maintenance of spatial attention. *NeuroImage*.

[59] Romei V., Rihs T., Brodbeck V., Thut G. (2008). Resting electroencephalogram alpha-power over posterior sites indexes baseline visual cortex excitability. *Neuroreport*.

[60] Kober S. E., Schweiger D., Witte M. (2015). Specific effects of EEG based neurofeedback training on memory functions in post-stroke victims. *Journal of Neuroengineering and Rehabilitation*.

