# Supplementary material for: Increased Alpha-Rhythm Dynamic Range Promotes Recovery from Visuospatial Neglect: A Neurofeedback Study
Source: Neural Plast. 2017 Apr 25;2017:7407241. doi: 10.1155/2017/7407241 (PMC5424484; doi:10.1155/2017/7407241)
Supplement: Supplementary file 1 — Figure S1. Individual structural MRI lesion maps of the 5 neglect patients. Figure S2. Experimental timeline for each patient. Gold: Initial baseline assessment consisting of resting-state EEG and behavioral tests, followed by a wait-list period (Week 1). Brown: Pre-post baseline assessment flanking first and last session of NFB training. Green: NFB training only. Patients underwent 21 minutes of neurofeedback training on a daily basis (except weekends). Figure S3. Omission rates for the target cancellation test at the 5 different times of assessment (2nd and 3rd, as well as 4th and 5th are pre-to-post NFB measures). Omission rate is plotted for A) the entire search display (total), B) center, C) left , and D) right side. [file 7407241.f1.docx]

**Supplementary Results**

**
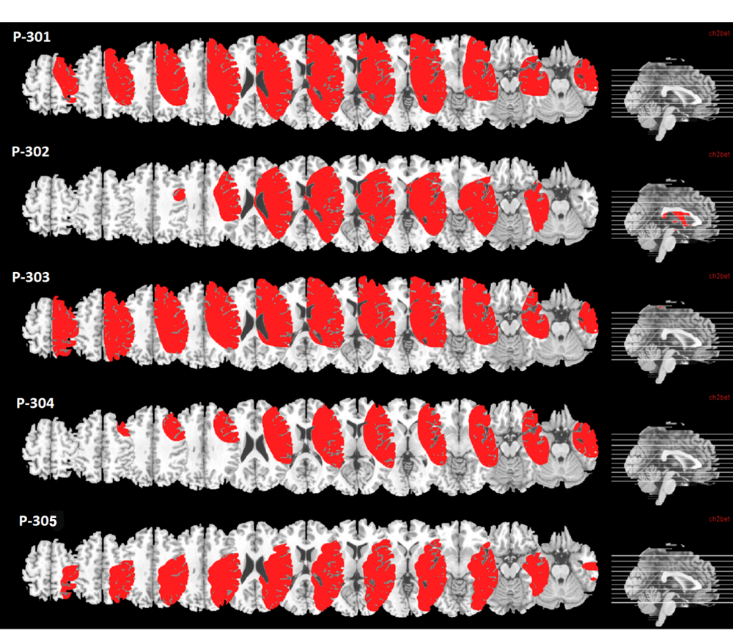
**

***Figure S1:*** *Individual structural MRI lesion maps of the 5 neglect patients*


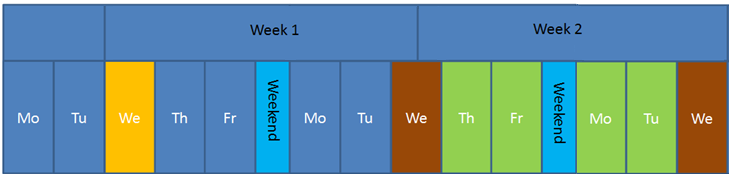


***Figure S2.*** *Experimental timeline for each patient.* ***Gold****: Initial baseline assessment consisting of resting-state EEG and behavioral tests,* *followed by a wait-list period (Week 1).* ***Brown****: Pre-post baseline assessment flanking first and last session of NFB training.* ***Green****: NFB training only. Patients underwent 21 minutes of neurofeedback training on a daily basis (except weekends).*

B


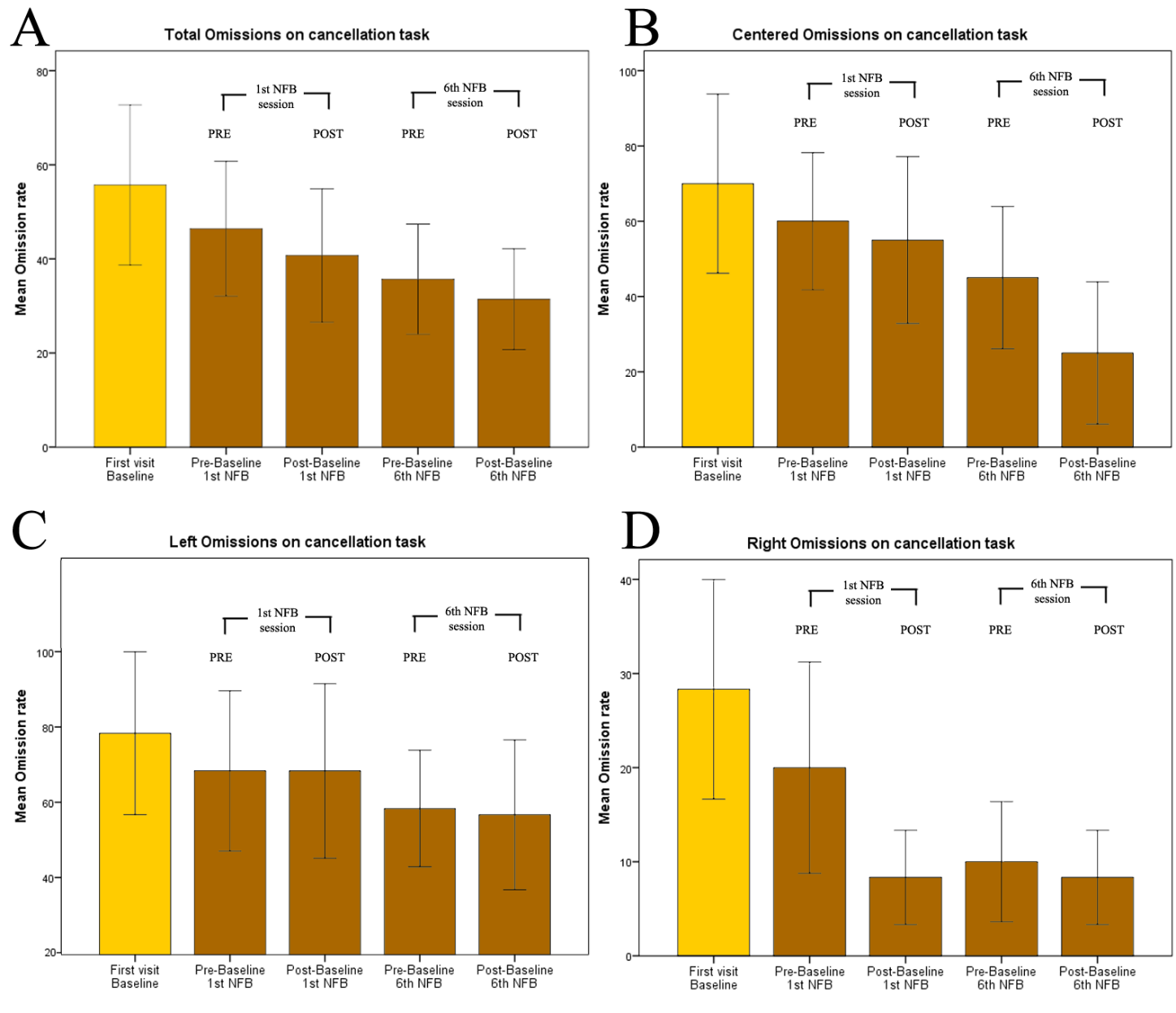


***Figure S3:*** *Omission rates for the target cancellation test at the 5 different times of assessment (2nd and 3rd, as well as 4th and 5th are pre-to-post NFB measures). Omission rate is plotted for* ***A)*** *the entire search display (total),* ***B)*** *center,* ***C)*** *left , and* ***D)*** *right side.*

**Short- and long- term modulation of visuospatial attention**

Two-way ANOVAs were used to test for short- and long-term effects between the wait-list period and that of the NFB intervention, yielding no significant interactions on cancellation task performance (p > 0.05). As seen in Fig S3, short-term modulation was defined as changes pre-post NFB, while long-term differences were calculated between error rates at the first wait-list visit (one week before NFB) and the baseline score preceding the last (6^th^) NFB session.

It is important to highlight that this negative finding may partly stem from the very low sample size (5 subjects), which reduces the statistical power for detecting significant differences. If we look at the longitudinal performance over time, we notice that patients tended to reduce their error rate stepwise for the cancellation and bisection tasks (Fig S2). However, paired sample t-tests did not uncover significant within-group changes for any of the performance measures (p > 0.05), either in the short- or the long-term.

**EEG neurofeedback technique**

We used a single-channel ProComp+ amplifier (Thought Technology, Canada) interfacing with EEGer 4.2 neurofeedback software (EEG Spectrum Systems, CA). Feedback was provided from the scalp electrode P4 (overlying right posterior parietal cortex), while ground and reference electrodes were placed on the right and left earlobes, respectively. Each NFB session started with a 3 minutes resting-state condition (without feedback), during which patients were asked to relax with their eyes open and gaze at an empty screen. This was followed by 21 min of NFB, composed of 7 runs of 2:50 minutes of continuous training, and 10 seconds of rest, during which the patient’s regulation score was displayed (see below).

In order to conduct the online NFB training, the EEG signal was band-pass filtered using IIR (infinite impulse response) to extract alpha (7–13 Hz) amplitude with an exponential moving average epoch size of 0.5 seconds. The protocol reinforcement schedule was set to give the patient a reward based upon the suppression of their absolute alpha amplitude at the location of the feedback electrode (P4). In the start of each training session, the threshold amplitude for reward was set manually at a position that would provide circa 60% temporal occurrence of reward, based on the initial 3 minutes of baseline average. In case of participants achieving disproportionately large (more than 90%) or lower (less than 40%) reward rates during the NFB training session, the reward rate was manually re-applied by the experimenter at the beginning of the following (3 min) period, based on the signal of the preceding 30 seconds. After every 3-minute training run, a regulation score was displayed, which was computed as a proportion of the cumulative amount of time during which the alpha amplitude was kept below its training threshold (1 second = 1 point).

The visual feedback was displayed on a 20” monitor via a “Space Race” game that includes several representations of EEG control, including a bar graph and a racing space-ship. This information depicted the patient’s ongoing performance and was proportional to the real-time alpha amplitude fluctuation measured at P4. Patients were told that the space ship would move forward through space when their brain was “in the good zone” of the target neural activity, thus increasing their points in the game (i.e. alpha lower than threshold), and that otherwise the space ship would stay stationary if the brain was “out of the good zone” (i.e. alpha higher than threshold). The space ship was shown at the center of the screen and its motion rendered by displacement of stars in the peripheral visual field bilaterally (i.e. stars in space), thus ensuring that EEG measurements and feedback signals were not contaminated by changes in eye fixation or saccades to the left or right side. The aim of the training was for the participants to learn to keep the space ship travelling forward thanks to the provided feedback. If the patient was not performing well, additional cues on the possible strategy were given between NFB runs, such as “try to focus on something you see” or “try to imagine things in your left field of vision”.

To quantify how well patients performed during NFB, the EEG recording during NFB of each patient was separated in 7 runs (one for each 3 minute training run). The mean alpha-band amplitude for each run was then divided (normalized) by the 3-minute resting-state amplitude preceding the NFB training (referred to as ‘NFB training % alpha change’). Similarly, we computed a ratio for the resting-state alpha change, whereby mean amplitude of the second baseline was divided by the mean amplitude of the first baseline (referred to as ‘resting % alpha change’).
